# Influence of Nanotechnology and the Role of Nanostructures in Biomimetic Studies and Their Potential Applications

**DOI:** 10.3390/biomimetics2020007

**Published:** 2017-05-26

**Authors:** Puneet Garg, Prerana Ghatmale, Kirtan Tarwadi, Sachin Chavan

**Affiliations:** Department of Nanotechnology, Bharati Vidyapeeth Deemed University, College of Engineering, Pune, MH 411043, India; prerana_ghatmale@outlook.com (P.G.); kirtanvt@gmail.com (K.T.); sschavan@bvucoep.edu.in (S.C.)

**Keywords:** biomimetics, biomimicry, nanostructures, nanotechnology, nature

## Abstract

With the advent of nanotechnology, by looking further deep down into the molecular level, today, we are able to understand basic and applied sciences even better than ever before. Not only has nanoscience and nanotechnology allowed us to study the composing structures of materials in detail, it has also allowed us to fabricate and synthesize such nanostructures using top-down and bottom-up approaches. One such field, which has been significantly influenced by the dawn of nanotechnology is biomimetics. With powerful spectroscopic and microscopic tools presenting us with images like double nanostructured pillars on the lotus surface for superhydrophobicity, the conical protuberances of moth eye demonstrating anti-reflection properties and nanostructured spatulae of gecko feet for high adhesivity, we are now able to fabricate these structures in the lab with properties showing close resemblance to their natural counterparts. Here, we present a review of various nanostructures that exist in nature, their fabrication techniques and some of their promising future applications. We hope this review will provide the reader with a basic understanding of what biomimetics is and how nanotechnology has significantly influenced this field.

## 1. Introduction

Biomimetics or biomimicry is one of the most intriguing fields of the contemporary world. In layman terms, it means the transfer and implementation of abstract principles and concepts from living nature to fulfil our requirements of obtaining better materials and efficient machinery. From the designing of aircraft by studying the flight of birds [1] to manufacturing photonic integrated circuits by studying wing patterns of a butterfly [2], the field of biomimicry has always caught man’s attention. The solution to some of the most complex material fabrication problems and the design of system models already exist in nature and can be duplicated by looking more closely at the structures and patterns present in nature. This is what biomimetics does. It involves the study of how nature works, its models, various processes by which those models are executed and the different elements constituting the model.

Delving deep into the history of the subject, it has been a remarkable journey for bioinspired studies. From the old records of Leonardo Da Vinci studying bird flight [3], to the progress made by Otto Lilienthal by deriving inspiration from white stork flight to make objects fly that are heavier than air [4], biomimetics have come a long way since it first originated. Designing electronic circuits like the Schmitt trigger by studying nerve propagation in squids, the fabrication of novel materials with properties like self-healing [5], self-cleaning and superhydrophobicity [6], the synthesis of super glues and adhesives [7] and anti-reflection coating technologies [8] are all a fruitful result of biomimetics research.

In this review, we will be focusing on the nanostructures present in nature that have inspired the design and synthesis of novel materials. The points that we consider in this review are: (1) the biomimetic material does not contain any part directly obtained from nature and is artificially fabricated; and (2) the nanoscale must be regarded as the base while designing such a biomimetic material. To further elaborate on these points, here, we exclude the studies where natural moieties were altered in some way to make them function according to our needs, and this procedure was referred to as biomimetic approach. An example of this method includes functionalization of deoxyribonucleic acid (DNA) for obtaining novel properties [9]. Additionally, we also exclude studies where the design as a whole was influenced from any natural models or processes and was not directly inspired from nanostructures of its material. For example, we exclude the design of planes being inspired by bird flight (relating to differences in air pressure and movement of wings) [1], research in the diving behavior of animals to study diving in humans and other similar studies. It should also be noted that the biologically inspired nanostructures we review here are listed in no particular order; nor is this a complete list of such structures. As more nanostructures are being studied and incorporated into the literature, new biomimetic materials are being fabricated which will continue to have a major impact on human applications.

## 2. Role of Nanotechnology and Nanostructures in Biomimetics

It was only with the advancement in the field of nanotechnology that we were able to study the nanostructures present in nature. Since most of the wonders of nature take place at the molecular level, i.e., in the nanoscale, only when powerful analytical techniques like scanning tunneling microscopy (STM) [10] and atomic force microscopy (AFM) [11] were developed, were we able to look closely at the spectacular working of natural materials. For example, we were able to mimic gecko feet to fabricate materials in the lab with the dual characteristics of superhydrophobicity, as well as high adhesivity towards water [12], but only after their nanostructured spatulae were studied. Another famous example is that of a lotus leaf [13], which has been used to synthesize self-cleaning, superhydrophobic surfaces. This again happened only when double nanostructured pillars present on the surface of a lotus leaf were studied and mimicked. This superhydrophobic property is rightly called the “lotus effect”. Thus, we are now able to synthesize bioinspired materials in the lab by using various top-down and bottom-up nanomaterial synthesis techniques. The top-down approach involves consecutive cutting or slicing of bulk material to obtain smaller and smaller particles until nanosized particles are obtained (e.g., mechanical ball milling); whereas the bottom-up approach involves self-assembly of small precursor particles to obtain the desired nanomaterial (e.g., processes involving chemical synthesis). Examples relevant to the field of biomimetics include fabricating structures mimicking gecko feet by plasma enhanced chemical vapor deposition [14], chemical synthesis of artificial antennas—using bis(phenylethynyl)anthracene (BPEA), borondipyrromethene (BDPY) and zinc tetraarylporphyrin (ZTAP)—and reaction centers (using porphyrin, fullerene and carotenoid) for mimicking the process of photosynthesis in the lab [15] and utilizing the roller nanoimprint fabrication technique for fabricating moth eye-like conical protuberances [16]. Thus, in a nutshell, the development of sophisticated tools and techniques to uncover the secrets of nanostructures and their synthesis methods is what has led to tremendous progress in the field of nanotechnology, and this was responsible for contributing a substantial amount of progress in biomimetics, thus realizing its full potential.

## 3. Different Types of Biomimetic-Inspired Nanomaterials and Their Synthesis

Although there are a number of nanomaterials that have been artificially synthesized by mimicking the nanostructures present in nature, we will cover here some of the most important materials with great future application potential. We begin by looking at the double nanostructured pillars on the surface of a lotus leaf showing superhydrophobicity and study their design pattern for artificial synthesis. This is followed by the study of nanostructured spatulae of gecko feet and their dual characteristic of superhydrophobicity and high water adhesivity. We then look at how the biological wound healing process is mimicked to follow a synthetic self-healing process for producing self-repairing materials. Next, we jump over to flora, studying the process of photosynthesis in detail and its mimicry for the generation of hydrogen fuel. Going back to the animal kingdom once again, we look at the nanostructures of moth eye, butterfly wings and frog toe pads. Simultaneously, we also look at some of the fabrication techniques for these nanostructures and their potential applications.

### 3.1. The Lotus Effect

**Figure 1:**
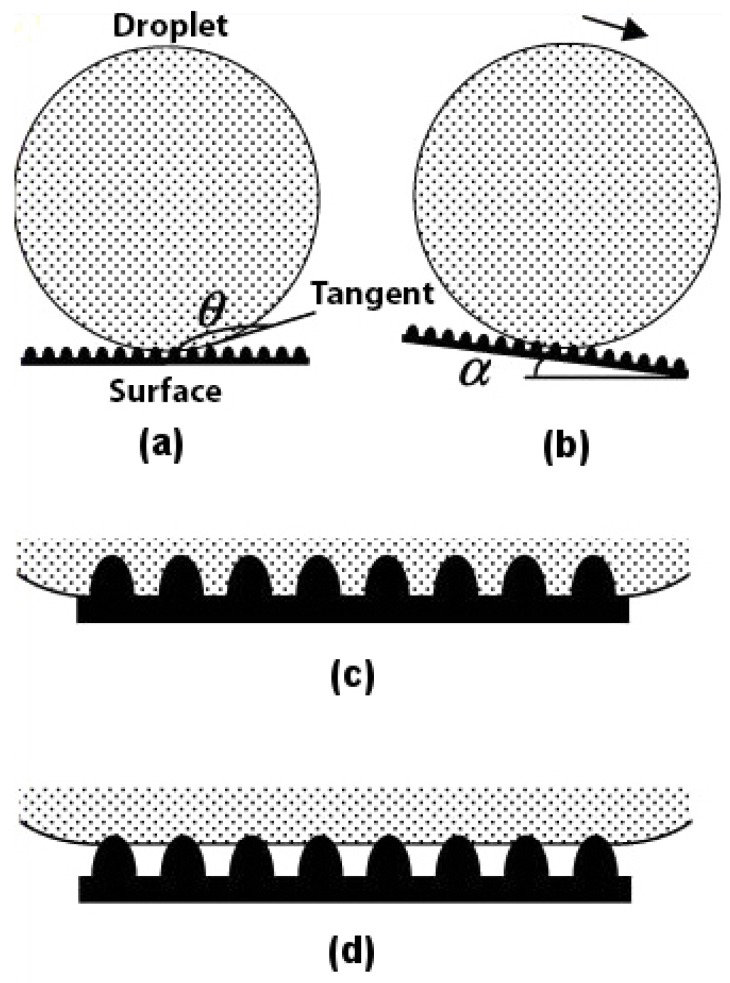
Water droplet on a rough surface. (**a**) Contact angle (θ); (**b**) Roll-off angle (α); (**c**) Homogeneous wetting; (**d**) Heterogeneous wetting. Adapted with permission from [17]. Copyright 2004, American Chemical Society.

Superhydrophobicity is based on the concept of contact angle and roll-off angle. For a surface to be called superhydrophobic, the contact angle (θ) between the water droplet and the surface should be as high as possible, and the roll-off angle (α) should be as low as possible, as shown in Figure 1a,b. Any rough surface can become wet in two ways. Firstly, if the liquid droplet penetrates completely into the rough surface to reach its troughs, it is known as homogeneous wetting; see Figure 1c. Secondly, if the troughs of the rough surface have any other fluid or air trapped inside them, then there is heterogeneous wetting; see Figure 1d.

The type of wetting is a major contributing factor to the superhydrophobicity of the material. It has been found [17] that the lotus leaf follows a heterogeneous type of wetting. The reason why nature prefers heterogeneous wetting over homogeneous wetting is because in the former, the wet area is always less as compared to the latter, as shown in Figure 1c,d. This ensures that the contact angle for a water droplet on the surface of a lotus leaf following the heterogeneous wetting regime is greater and not lower, as in the case of the homogeneous wetting regime. This makes for an easier roll-off of the water droplet from the lotus leaf and, thus, has a lower roll-off angle. The type of wetting plays such an important role in superhydrophobicity that if water is made to condense in the troughs of a lotus leaf [18], the surface of the leaf starts demonstrating a homogeneous wetting character, thus becoming hydrophilic with a contact angle much lower than 90.

**Figure 2:**
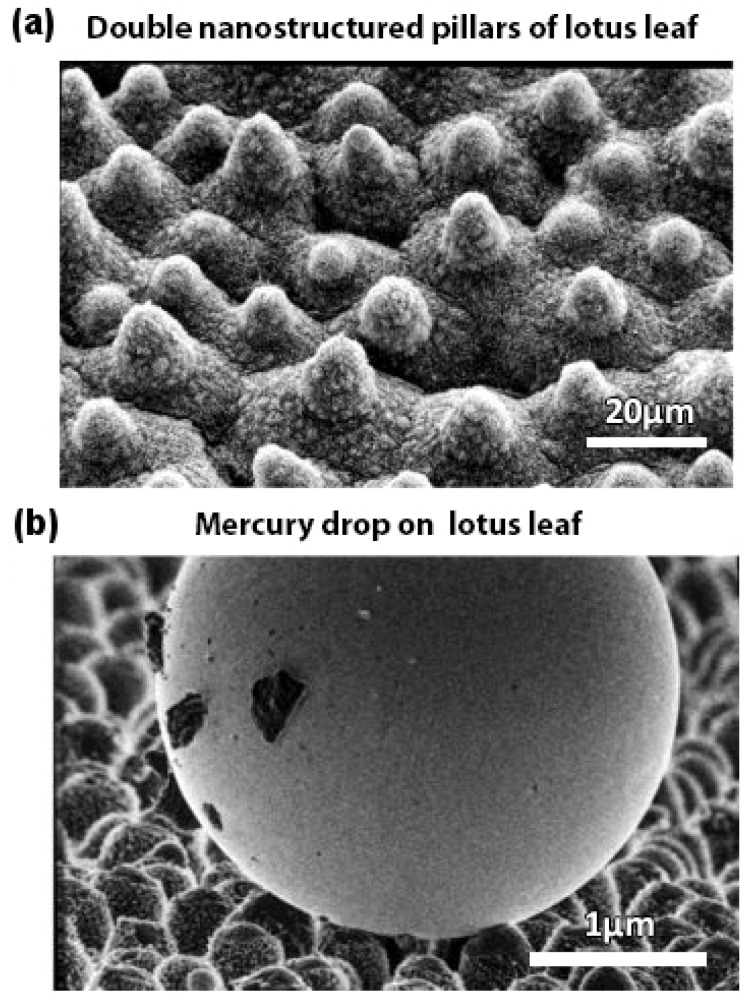
Lotus effect. Scanning electron microscopy (SEM) images of (**a**) double nanostructured pillars on the surface of a lotus leaf and (**b**) composite mercury drop on top of the rough surface of a lotus leaf making a large contact angle. Adapted with permission from [20]. Copyright 2004, American Chemical Society.

**Figure 3:**
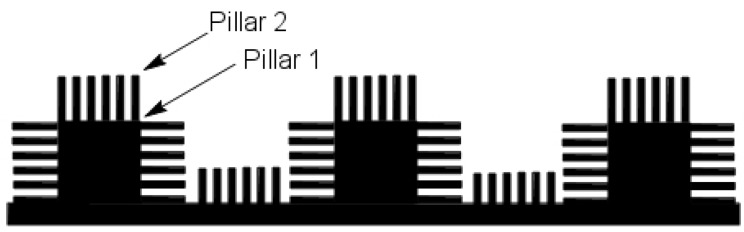
Biomimetic design inspired from the double nanostructured pillars on the surface of a lotus leaf.

Barthlott and Neinhuis [19] first collected data on how dirt-contaminated lotus leaves self-cleaned themselves due to their superhydrophobic characteristics. It was years later that the researchers found that it was the effect of surface roughness and the wetting regime of the lotus leaf surface that resulted in the self-cleaning effect. Furthermore, it was found [20] that the surface roughness of a lotus leaf was due to multiple nanostructured pillars (Figure 2a) (with sizes in the range of 200 nm to 1 μm) on its surface. Figure 2b illustrates a drop of mercury on the surface of a lotus leaf. Thus, it was now possible to fabricate a design that would mimic the superhydrophobic behavior of a lotus leaf. A double-pillared structure design as shown in Figure 3 is found to be best suitable for fabricating superhydrophobic self-cleaning surfaces by greatly amplifying the contact angle.

The double pillared nanostructures can be artificially fabricated using the soft lithographic imprinting technique [21]. In this method, the lotus leaf is used as a natural template, and a liquid polymer is cast on the surface of this lotus leaf. When solidified, this polymer layer is peeled off to form a negative template. Another layer of the same or a different polymer is then cast on this negative template and peeled off in a similar fashion to transfer the nanostructures, mimicking the surface morphology of the lotus leaf on this second layer. Many polymers like poly(dimethylsiloxane) (PDMS) [22,23], poly(vinyl chloride) (PVC) [24] and poly(ethylene oxide) (PEO) [25] have been used to form negative templates and thus, superhydrophobic surfaces.

Much work has been done in the field of superhydrophobic surfaces in the last decade. However, still, we do not see them readily available in the market. This is because, although superhydrophobic surfaces have been fabricated in the lab with very high contact angles and very low roll-off angles, these are not durable enough to be commercialized. They are susceptible to dynamic impact, mechanical deterioration, environmental and chemical degradation and organic or bacterial contamination. Moreover, the tests that are conducted for durability by different researchers are also not standardized and, thus, fail to support industrial-level manufacturing of superhydrophobic surfaces. Inevitably, today, the area of interest in this field is the fabrication of superhydrophobic surfaces with high durability [26]. Furthermore, as these coatings are implemented in areas where they are subjected to various kinds of environments, additional properties like anticorrosion, antifouling, self-healing capabilities, UV-B blocking, flame retardancy and oil repellency are actively researched [27,28].

### 3.2. Gecko Feet

Gecko lizards are found throughout the world in places with warm climates. They are of particular importance due to their feet, which allow them to climb any surface whether smooth or rough, inverted or vertical, without any problem. This characteristic feature of gecko feet results from their unique microstructured setae (3–7 μm in diameter and 20–70 μm in length), which are keratinous hairs present in millions on the surface of their feet, and each seta is further subdivided into hundreds of nanostructured spatulae (100–200 nm in diameter). The setae are superhydrophobic in nature, making a contact angle of 160 with water [29]. However, it has also been found that not only these setae showed superhydrophobicity, but also an adhesive property towards water. This is a very unique dual characteristic behavior of gecko feet, which make them superhydrophobic, as well as highly adhesive towards water at will. The adhesion property is temperature-dependent as observed by Niewiarowski et al. [30], who found that adhesion of gecko feet towards a water droplet was halved at 32 C than at 12 C.

**Figure 4:**
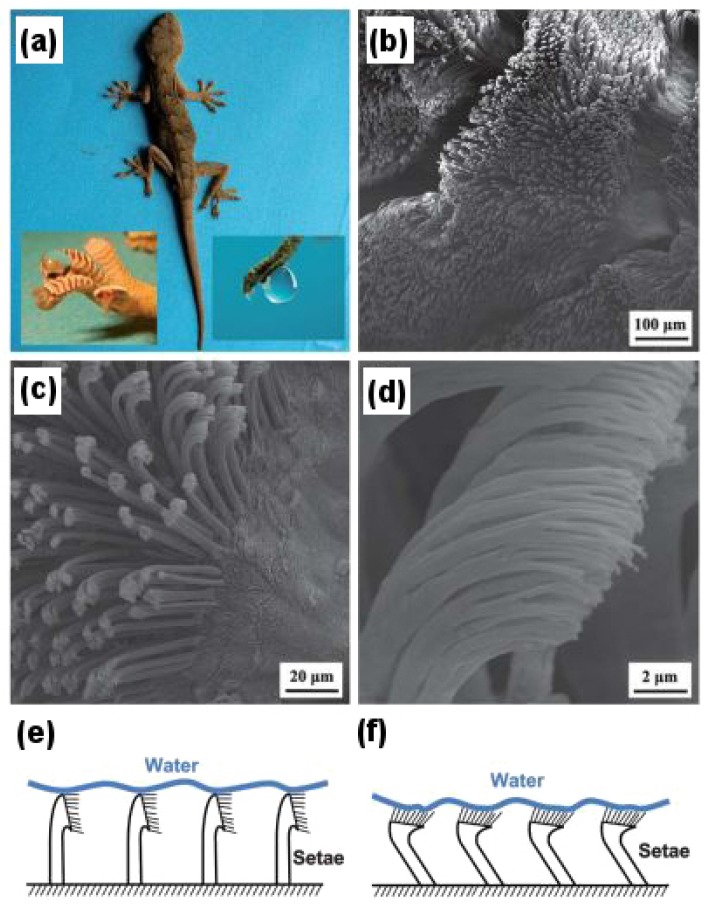
High-adhesion superhydrophobic surface of gecko feet. (**a**) Gecko lizard; the inset on the left shows the superhydrophobic character, and the inset on right shows the highly adhesive characteristic of gecko feet; (**b**) Scanning electron microscopy (SEM) image of the gecko foot at low magnification; (**c**) High magnification image of the gecko foot with visible microstructured setae; (**d**) nanostructured spatulae branching out from single seta in a high resolution SEM image; (**e**) Schematic of the superhydrophobic character of gecko setae with the water droplet; (**f**) Schematic of the highly adhesive character of gecko setae with the water droplet. Reproduced from [32] with permission of The Royal Society of Chemistry.

Figure 4 demonstrates the microstructures and nanostructures present on the surface of gecko feet and their characteristic features in detail. The inset to the left of Figure 4a shows a drop of water almost perfectly spherical in shape on the foot of an anesthetized gecko revealing its superhydrophobic character, whereas the inset to the right demonstrates how a water droplet clinches to the surface of the gecko foot even when it is turned 90, showing its highly adhesive behavior towards the droplet. Figure 4b–d shows scanning electron microscopy (SEM) images of setae and spatulae, whereas Figure 4e,f illustrates the schematic as to how setae and spatulae are responsible for providing a superhydrophobic, as well as highly adhesive dual character to gecko feet. Autumn and Hansen [31] first demonstrated the superhydrophobicity of setae by isolating them on a glass substrate and mimicking the arrangement as shown in Figure 4e. However, to demonstrate its highly adhesive behavior due to the very large number of nanopillared spatulae coming in contact with the water droplet, the arrangement as shown in Figure 4f can be used.

Such a high adhesive force of gecko feet is said to be the result of van der Waals interactions [29,33] as in the case of solid–solid adhesion, but here, it is between the water droplet and the numerous nanopillared spatulae. It has also been speculated that the actual contact area between the water droplet and spatulae is much larger than we predict [34], which provides the gecko feet with this kind of highly adhesive behavior. The role of capillary-induced adhesion also cannot be ruled out [35].

Liu et al. [32] fabricated superhydrophobic, as well as highly adhesive polyimide films with densely packed microclusters and nanopillars mimicking gecko feet via anodic aluminum oxide (AAO) templates. A value of about 66 ± 3 μN was obtained for the adhesion force between liquid–solid (i.e., water droplet–polyimide film) measured using the highly sensitive microelectromechanical balance system. Qu and Dai [14] mimicked gecko feet using vertically aligned single-walled carbon nanotubes (VA-SWCNTs), which they synthesized using the plasma enhanced chemical vapor deposition (PE-CVD) technique and a fast heating process. The micro- and nanostructures mimicking gecko feet fabricated using this technique also demonstrated excellent thermal and electrical properties making it possible for them to be used at higher temperature (∼125 as compared to scotch tape and super glue) and in semiconductor devices, respectively.

More recently, inspired from the self-cleaning capabilities of gecko feet without the use of water, focus has been shifted to the fabrication of artificial dry self-cleaning membranes [36]. Furthermore, due to their highly adhesive nature, gecko feet-inspired mimics have demonstrated their use for the pick up and drop off microparticles, which has further led to the manufacturing of complex micropatterns. This property of gecko feet-inspired artificial adhesives is now being used in many potential applications like in micromanipulation for the fabrication of microelectromechanical systems (MEMS) and in biomedical devices [37].

### 3.3. Self-Healing

The skin that covers the body of all vertebrates is composed of two main layers: the epidermis (outer layer) and the dermis (inner layer) [38]. In case we sustain an injury, these two layers of skin tear apart or are removed, exposing internal body tissues to the environment. This sets in the natural process of wound healing by our body, which forms the basis of our next biomimetic approach. To grasp the concept of self-healing in materials, it is essential to discuss the process of wound healing in belief, which is done next.

**Figure 5:**
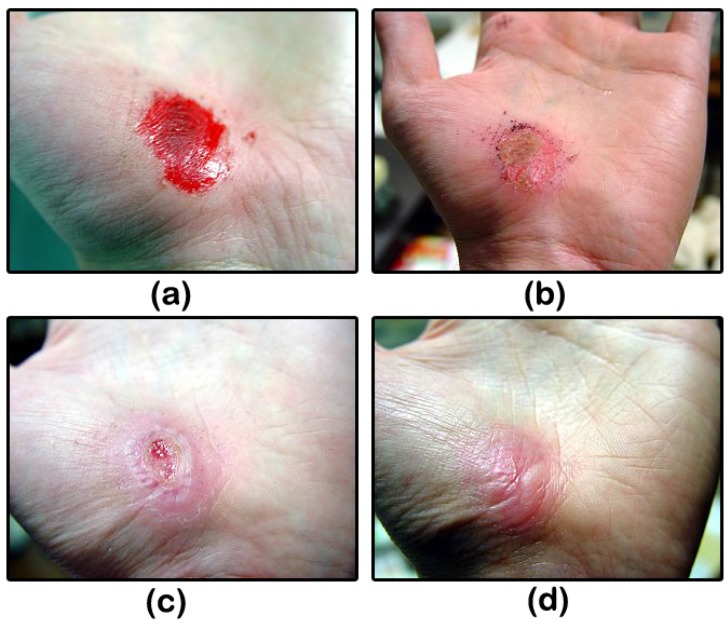
Skin wound healing. Hand abrasion (**a**) on the day of injury; (**b**) three days after injury; (**c**) 17 days after injury; and (**d**) 30 days after injury.

The wound healing process takes place in three steps: (i) hemostasis or blood clotting and inflammation; (ii) proliferation or tissue growth; and (iii) maturation or tissue remodeling [39]. Hemostasis is the cessation of blood flow at the site of injury to prevent blood loss. This happens by first sticking of blood platelets at the injury site, which then takes an amorphous shape to further promote clotting. Simultaneously, a chemical response is generated and sent, activating fibrin, which glues all of these platelets together to form a clot that stops the blood flow [40,41]. After this, the white blood cells engulf all of the bacteria and pathogens along with dead and damaged cells that are present at the injured site by a process known as phagocytosis [42]. This happens during the inflammation phase. In the next step, i.e., proliferation, new blood vessels form [43] along with the structural support provided by extracellular matrix [44]. Epithelial cells proliferate, as well providing cover for the newly formed tissue [45], and finally, wound contraction ensures decreased wound size. In the third and last step, collagen is realigned along wound boundaries, and unneeded cells undergo programmed cell death or apoptosis. Figure 5 presents a pictorial representation of the wound healing process.

**Figure 6:**
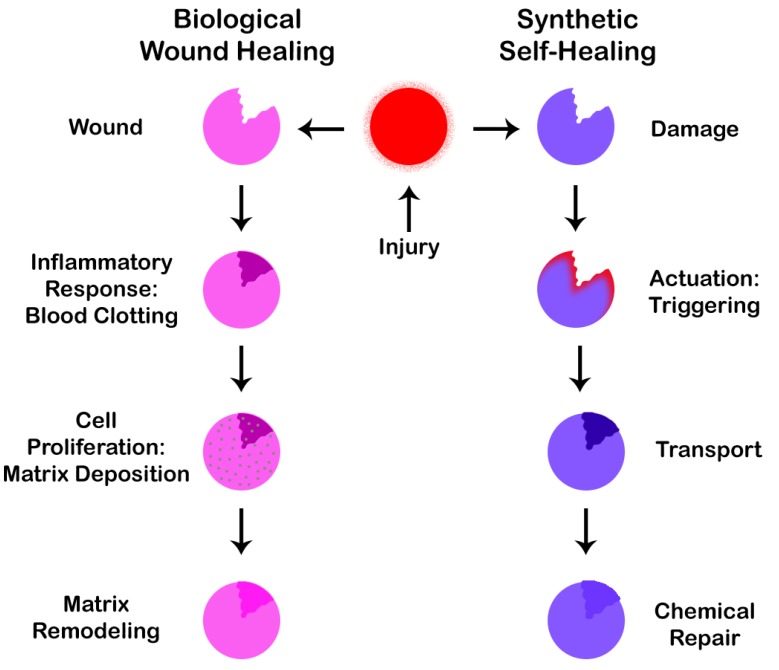
Similarities between the biological wound healing process and the synthetic self-healing process.

Inspired by the self-healing capabilities of the human body, we are on our way to create smart materials that are able to self-heal based on a process similar to wound healing. Figure 6 shows demonstrates similarities between the two processes of biological wound healing and synthetic self-healing. As can be seen from the figure, the two processes are strikingly similar in nature.

In synthetic self-healing, the first step is an actuation or triggering mechanism, which takes place if a crack develops. This is followed by a second step in which the material is transferred to the affected site leading to a third and final step of carrying out the repair operation of the affected area as per the healing mechanism applied. There are a number of healing mechanisms that can be implemented as discussed in subsequent paragraphs. It is also worth noting that not only humans and animals, but plants [46] also demonstrate self-healing capabilities by fighting against infection from pathogenic plants and preventing themselves from desiccation [47], i.e., extreme dryness.

Different types of materials like polymers and their composites [48], cementitious materials [49], ceramics [50] and metals [51] are being actively researched to provide them with self-healing capabilities. For example, smart polymeric materials possessing self-healing capabilities are being synthesized in three different ways, namely: capsule-based healing, vascular self-healing and the intrinsic approach to self-healing [48]. Various other techniques are continuously being discovered for materials to have self-healing properties.

**Figure 7:**
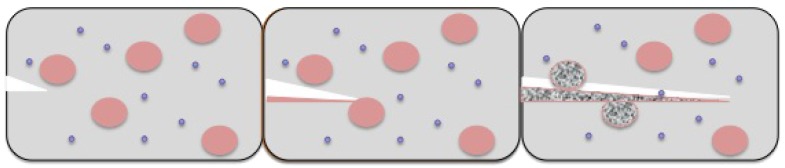
Self-healing in polymeric materials using the capsule-based approach.

In the capsule-based healing approach, the healing agent is packed in tiny capsules. As the crack or damage propagates and reaches these encapsulations, they rupture, releasing the healing agent into the crack, which repairs the damage. This is however a one-time repair mechanism, as once ruptured, the capsule is useless if the crack reappears at the same spot. In the vascular self-healing process, hollow tubes and capillaries are filled with healing agent and are connected in a network. This system of healing agent-containing capillaries can form one-, two- or three-dimensional networks, and their density depends on the amount of cracks, which can develop in the material over time. Once a crack propagates to these channels, the capillaries break, releasing the healing agent and repairing the crack. A major advantage to this approach is that it is a multiple time healing process. If a crack reappears at the same spot, capillaries releasing the healing agent can once again repair the crack. Empty capillaries from previous repair cycles can be refilled with healing agent externally or from other capillaries connected in the network. In the intrinsic type self-healing, there is no healing agent, rather a latent characteristic of the material, which promotes self-healing. This characteristic can be attributed to the chemical bonding within the material and physical interactions in the crack interfaces. Intrinsic self-healing is based on reversible reactions, like hydrogen bonding, ionic interactions, reversible polymerization, etc. Yet, an external trigger is often required, like heat, mechanical stress and electrical stimulus, to initiate the self-healing process [52]. Since reversible reactions are involved, this type of healing can occur many times at the same spot in case a crack appears. Figure 7 shows the self-healing process using the capsule-based approach.

In more recent studies, research is no longer focused on the refilling of gaps after crack development. Rather, emphasis is laid on self-healing capabilities of the various functionalities of the material [53]. These include self-repair of properties like resistance, conductivity and charge carrier mobility in conductors, emission, reflectivity and absorption in optoelectronic materials and various functions of novel films and coatings, like superhydrophobicity, flame resistance, surface energy, aesthetics and superamphiphobicity. Moreover, advanced trigger systems are also being researched, like stimulus by chemical, thermal, electrochemical, mechanical or photochemical impetus, as against the conventional crack propagation. In the coming years, hybrid self-healing materials will be the center of attention, which will involve an amalgamation of self-repairing capabilities for different properties of the material.

### 3.4. Photosynthesis

Photosynthesis is the process by which plants use carbon dioxide, water and energy to synthesize glucose, the building blocks of plants, and give away oxygen as the by-product. This process is represented by the equation as given below:

(1) $${6\mathrm{CO}}_{2}+6H_{2}O\overset{\mathrm{Light}}{\to }C_{6}H_{12}O_{6}+6O_{2}$$

Understanding the process of photosynthesis provides us with better insight into the working of plant mechanisms and helps us to reap fruitful results by its biomimicry. Subsequent paragraphs describe the process of photosynthesis in brief [54].

To carry out the photosynthetic process, plants obtain carbon dioxide from the atmosphere; water is derived from the ground through roots; and energy is obtained from the sun. Light from the sun contains photons from a wide range of the electromagnetic spectrum, but for plants, only photons in the range of the visible spectrum (400–700 nm) are required. To capture these photons, plants have special pigments called chlorophylls. The color of this pigment is a result of the light reflected. For example, reflecting green and yellow wavelengths of light and absorbing blue and red wavelengths are what give plants their characteristic yellowish-green color. The blue and red wavelengths of light are sufficient to provide the required energy to plants for photosynthesis.

**Figure 8:**
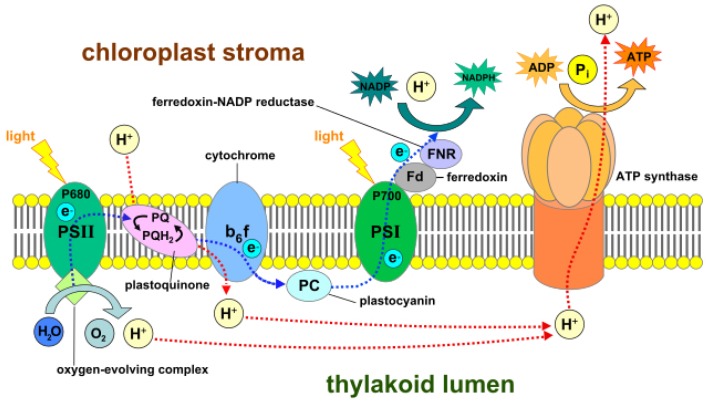
Light-dependent photosynthesis reactions demonstrating the mechanisms of photosystem-I (PSI), the cytochrome b_6_f complex, photosystem-II (PSII), ferredoxin-NADP^+^ reductase (FNR) and ATP synthase. ADP: Adenosine diphosphate; ATP: Adenosine triphosphate; NADP^+^/NADPH: Nicotinamide adenine dinucleotide phosphate.

**Figure 9:**
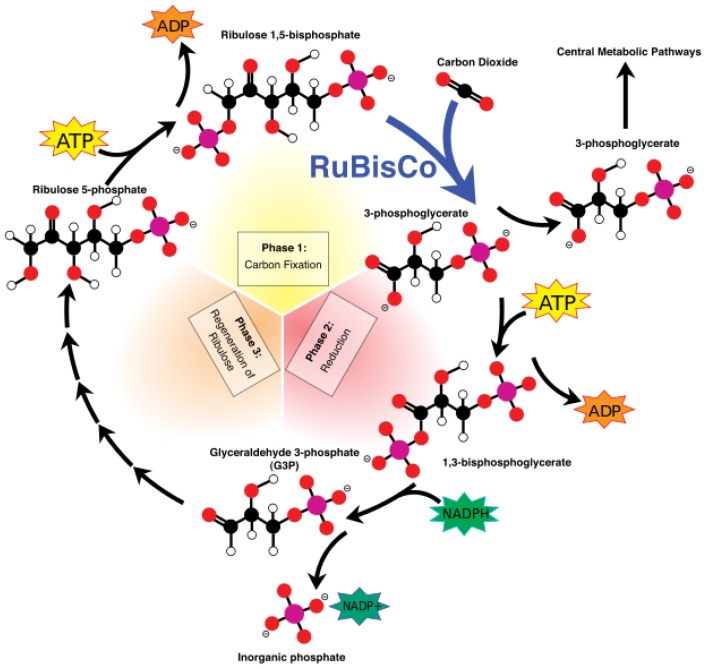
The Calvin cycle. Phase 1: Carbon fixation by ribulose-1,5-bisphosphate carboxylase/oxygenase (RuBisCO); Phase 2: Reduction; Phase 3: Regeneration of ribulose-1,5-bisphosphate.

Inside autotrophs, i.e., self-feeding plants, photosynthesis is carried out in special structures of the plant cell called chloroplasts. Photosynthesis is a two reaction set mechanism: (i) light-dependent reactions [55] and (ii) the Calvin cycle [55], as depicted in Figure 8 and Figure 9, respectively. Chloroplasts contain small disc-like structures called thylakoids [56], which are surrounded by colorless fluid stroma. Light-dependent reactions occur in thylakoid, whereas light independent reactions or the Calvin cycle occur in stroma. Thylakoid converts light energy from the sun to chemical energy, which ultimately drives the synthesis of glucose. Each thylakoid contains a pair of photosystems called photosystem-I and photosystem-II, which work together to achieve this goal. These photosystems contain chlorophyll molecules, which capture incoming photons of light, thereby exciting their electrons to a higher state. These excited electrons are emitted and channeled to a single chlorophyll molecule called the reaction center chlorophyll. Photons strike both photosystems simultaneously.

In photosystem-II, the reaction center transports the excited electrons via an electron transport chain. The electron transport chain is a group of proteins situated on the thylakoid membrane. In case the photosystem-II loses any excited electrons, they are replaced by a process called photolysis in which water is oxidized to produce free electrons and oxygen gas as a by-product. The high energy electrons in the electron transport chain are used to pump hydrogen ions from the stroma to the thylakoid via the cytochrome b_6f_ complex enzyme. This develops a concentration gradient between the stroma and thylakoid membrane, which is used to power a protein called ATP synthase that converts adenosine diphosphate (ADP) to adenosine triphosphate (ATP). The low-energy electrons remaining in the electron transport chain are pushed to photosystem-I where they are re-energized and again carried via a second electron transport chain to ferredoxin-NADP^+^ reductase (FNR), an enzyme that mediates the transfer of electrons from reduced ferredoxin to nicotinamide adenine dinucleotide phosphate (NADP^+^) for production of NADPH.

The ATP and NADPH molecules produced and released continuously in the stroma during these light-dependent reactions power the Calvin cycle, which is a set of light-independent reactions reducing carbon dioxide to produce carbohydrate glyceraldehyde 3-phosphate (G3P). The Calvin cycle is a three-step process consisting of: (i) carbon fixation; (ii) reduction; and (iii) regeneration of ribulose-1,5-bisphosphate (RuBP). During carbon fixation, one molecule of CO_2_ combines with one molecule of RuBP to produce two molecules of 3-phosphoglycerate (PGA). Then, during the reduction, one molecule of ATP reduces one molecule of PGA to one molecule of 1,3-bisphosphoglycerate (1,3-BPG), which is further reduced by a single molecule of NADPH to one molecule of G3P. Thus, a single turn of the Calvin cycle produces two molecules of G3P. When the cycle runs three times, it produces six G3P molecules, five of which are used for the regeneration of RuBP in the third and final step of the Calvin cycle. The remaining G3P molecules once accumulated are then used to form glucose, glycerol and fatty acids.

Two molecules of G3P form one molecule of glucose 6-phosphate, which can get rid of its phosphate to combine with fructose and form sucrose. Thus, it take six turns for the Calvin cycle to produce one glucose molecule. Glucose 6-phosphate is also used to synthesize starch and cellulose. This completes our description of the process of photosynthesis as it is carried out in nature.

To bring about artificial photosynthesis in the lab, we need various components, like: (i) antennas that have the capability to capture incoming photons of light; (ii) a reaction center where all electrons pile up to be transported by an electron transport chain; and (iii) some kind of electron transport mechanism, which would lead to the development of the potential gradient for pumping protons.

It is now possible to synthesize artificial antennas that can harvest light [15]. These when linked with reaction centers are able to convert the excitation energy of photons to chemical potential by generating a long-lived charge separation. If however, the charge separation is short-lived, the potential energy of photons, which has been stored, does not last long enough to be harvested, thereby rendering the reaction center redundant. Artificial reaction centers when incorporated in a lipid bilayer of an artificial vesicular setup are responsible for generating transmembrane proton motive force by acting as light-driven proton pumps. This proton gradient generated is used by the ATP synthase enzyme to synthesize ATP, which is the desired high energy biofuel molecule.

**Figure 10:**
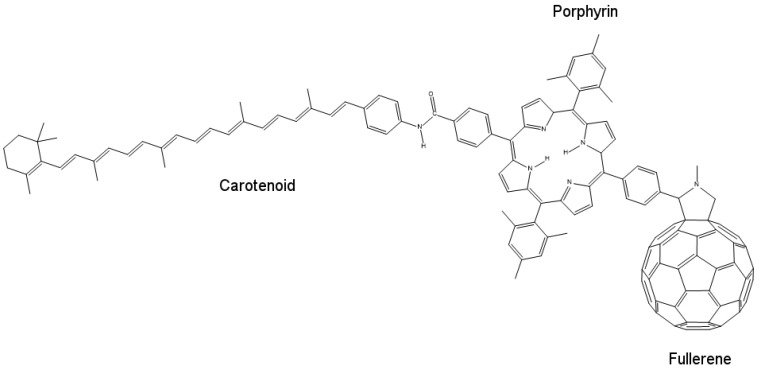
The molecular structure of an artificial reaction center, showing its constituent molecules porphyrin, fullerene and carotenoid.

To fabricate a model mimicking the photo-induced electron transfer mechanism or an artificial reaction center, we require an electron donor/acceptor chromophore, which is able to absorb visible light and can be bonded to another electron donor/acceptor moiety for generating the necessary charge generation. One such system can be designed using molecules of porphyrin, fullerene and carotenoid, as shown in Figure 10. Porphyrin, being less stable, mimics the chlorophyll present in plants. Fullerene (C_60_) behaves as the electron acceptor. Porphyrin (P) and fullerene when bonded covalently provide electronic coupling, which mimics the photo-induced electron transfer mechanism of the natural reaction center.

When excited by a photon of light, porphyrin and fullerene form a P^+^–$C_{60}^{-}$ charge separation pair, which recombines in 478 ps. This time interval is too short to harvest stored potential energy generated by this charge separation. To increase this time interval, electronic coupling between the two moieties should be weakened, which can be done by increasing the distance between donor and acceptor molecules, thus obtaining slow charge recombination. This is where carotenoid (C) plays its role. When carotenoid is linked covalently to porphyrin on another side, it completes the artificial reaction center model by overcoming the charge separation problem. Carotenoid increases the charge recombination time by 1000-fold to around 57 ns [57] acting as an electron donor in the process. When a photon strikes and charge separation occurs (C–P^+^–$C_{60}^{-}$), instead of recombining, this charge separation state forms another intermediate with carotenoid (C^+^–P–$C_{60}^{-}$), which then recombines very slowly, thus allowing the stored potential energy to be harvested.

To successfully mimic photosynthesis, however, only artificial reaction centers are not enough. They should work in combination with artificial antennas to fully develop a working model of an artificial photosynthetic system. An ideal artificial antenna would be able to absorb light and immediately transfer the electrons to constituent chromophores for generating charge separations. Sunlight is absorbed mostly by antenna systems and not reaction centers. Thus, it is extremely important to have excellent antenna systems to increase the efficiency of a lab-made, artificial photosynthetic system as much as possible. As natural photosynthesis can occur in a wide range of environmental and weather conditions, there are a number of antenna morphologies that exist in nature.

**Figure 11:**
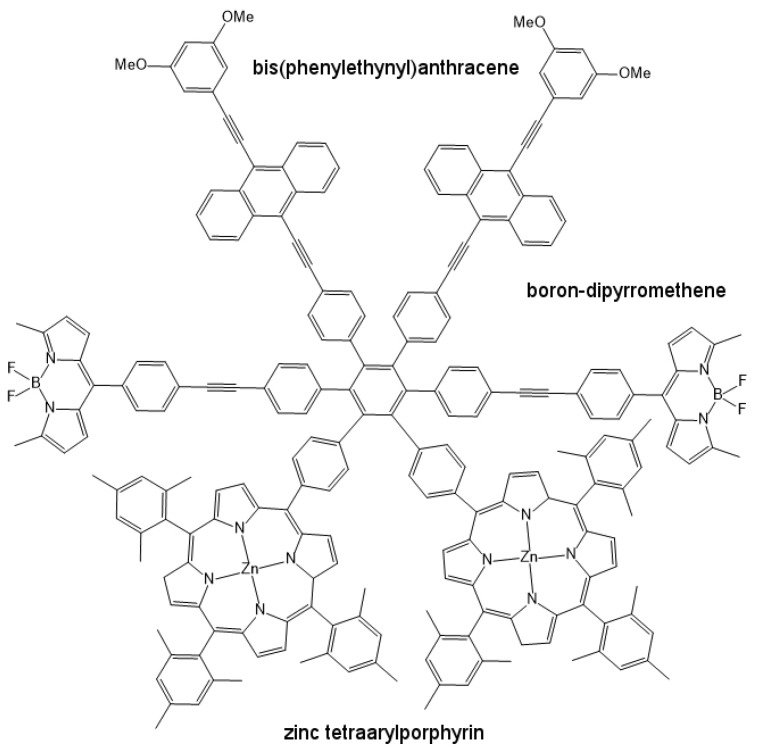
The molecular structure of an artificial antenna model, showing its constituent light absorbing molecules bis(phenylethynyl) anthracene (BPEA), boron-dipyrromethene (BDPY) and zinc tetraarylporphyrin (ZTAP).

One such type of artificial antenna morphology is illustrated in Figure 11 [58]. In this model, bis(phenylethynyl)anthracene (BPEA), boron-dipyrromethene (BDPY) and zinc tetraarylporphyrin (ZTAP) act as primary light absorbing components. BPEA absorbs strongly at around 450 nm, which represents the blue region of the electromagnetic spectrum; BDPY absorbs at 513 nm, which is close to green; and ZTAP absorbs at 418 nm (Soret region), and also at 557 nm and 598 nm, corresponding to the orange and red wavelengths of the electromagnetic spectrum, respectively. Thus, this model absorbs photons from almost the entire visible spectrum of light, which is usually the case with natural antenna systems. This antenna unit can then be attached to a charge separation unit like fullerene [57], which is a part of the artificial reaction center, thus completing the artificial photosynthetic system.

As an example of a working model of the proton pump [59], artificial reaction centers involving carotenoid–porphyrin–quinone and independent quinone molecules can be incorporated in the lipid bilayer, such that the quinone (Q) of carotenoid–porphyrin–quinone, being hydrophilic in nature, faces the external side of the lipid bilayer, whereas carotenoid being hydrophobic faces the inside of the membrane. The pump follows a mechanism of redox loop, in which, when excited by light, the reaction center forms a charge separation state C^+^–P–Q^−^. The quinone anion (Q^−^) of the artificial reaction center reduces independent quinone molecules to semiquinone anions, which can then grab a proton from the outside aqueous environment, becoming neutral themselves in the process. These neutral semiquinone molecules travel freely in the lipid bilayer, where, upon encountering the carotenoid ion (C^+^), they get reconverted into quinones, thereby releasing the proton in the interior side of the lipid bilayer. Many such carotenoid–porphyrin–quinone and quinone molecule pairs can transfer many protons simultaneously from the exterior of lipid bilayer membrane to its interior surface, thus generating a proton gradient, required for the synthesis of ATP.

Although we are now able to successfully fabricate artificial antennas and reaction centers to mimic the process of photosynthesis in the lab, there is always room for improvement. If we need more efficient hydrogen fuel generators, we have to increase the amount of light that is harvested by increasing the number of artificial antennas or by designing them more efficiently. This will ensure an increase in the amount of potential energy stored. Furthermore, the stored energy should be available to harvest for a longer duration of time, which can be achieved by increasing the charge separation period.

Some recent studies are a step in this direction. Artificial reaction centers based on porphyrin have been developed, which showed properties very similar to natural reaction centers of purple bacteria [60]. The charge separation distance in the reaction center of purple bacteria is around 7.0 Å, which was very closely mimicked in the artificial reaction center with a center-to-center distance of 6.2 Å. The electron-transfer reaction distances were also close, indicating the similarity between the electron-transport properties of the natural and artificial reaction centers. Moreover, the primary and secondary charge separation and recombination times were also strikingly similar, expect for the secondary charge recombination time, which was on a microsecond time-scale instead of the millisecond time-scale of the natural counterpart. Yet, further improvement is expected in the coming years. Other more advanced methods are now being researched, which involve the use of semiconducting photoelectrodes [61] or photobioelectrochemical systems with enzymatic cathodes and photoanodes [62] for mimicking photosynthesis and for fabricating photosynthetic systems on a large scale.

### 3.5. Moth Eyes

**Figure 12:**
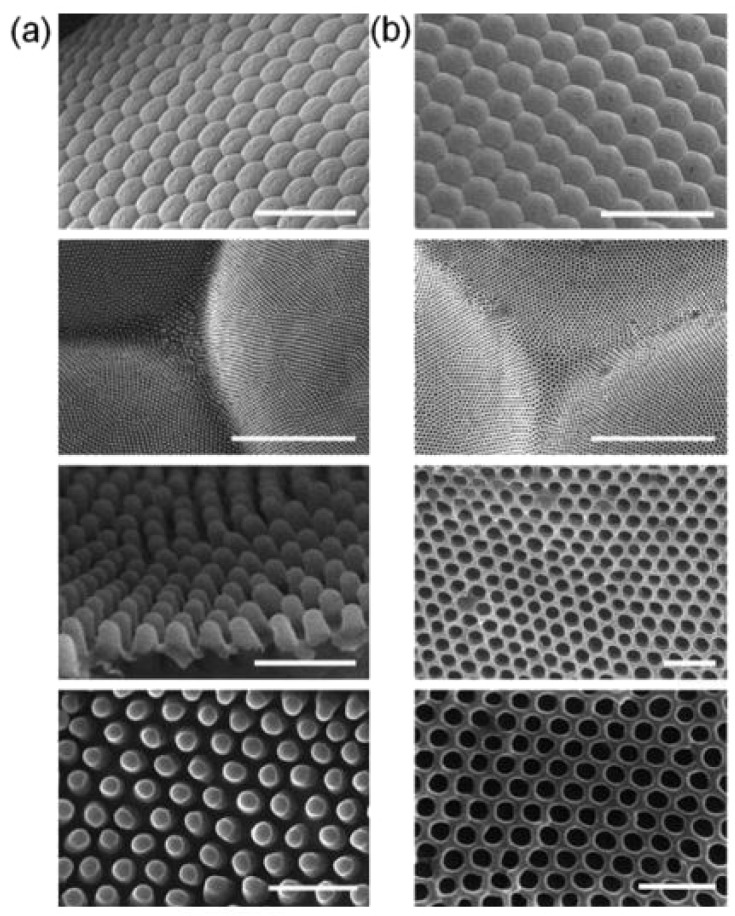
Anti-reflective structures of the moth eye and fabrication of biomimetic moth eye structures. Scanning electron microscopy (SEM) images of (**a**) various views of *Attacus atlas* moth eye demonstrating the conical protuberances on its surface and (**b**) similar views of the negative mold of moth eye produced using perfluoropolyether (PFPE), which can then be used to fabricate artificial moth eye-like nanostructures using the soft imprint lithographic method. Scale bars: first row images = 100 μm, second row images = 5 μm, and third and fourth row images = 500 nm. Reproduced from [65] with permission of The Royal Society of Chemistry.

Another interesting type of nanostructure for bioinspiration are the eyes of a moth insect. Bernhard [63], first in 1967, observed a unique characteristic of these nocturnal flying insects, where they played a game of camouflage with their predators by reducing reflection of light from their corneas. Later it was discovered that this anti-reflecting behavior was due to the regular arrangement of conical protuberances, which covered the entire surface of their eye. This anti-reflection property can be mimicked in the lab if the spacing between protuberances is much less than the incident wavelength of light, whereas the depth is significantly larger. A similar principle is applicable in acoustics as in the case of anechoic chambers. For fabricating artificial moth eye-like structures, this spacing between protuberances and the depth was found to be around 200 nm. Figure 12a depicts conical protuberance nanostructures of the moth eye.

**Figure 13:**
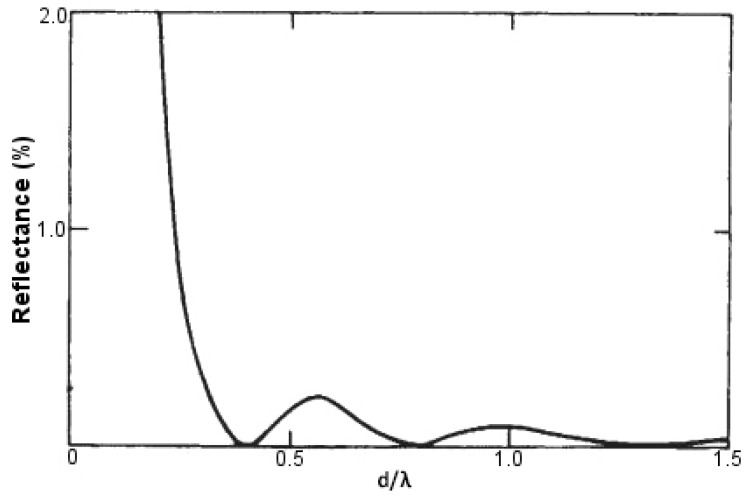
Computed dependence of the reflectance levels in fabricated moth eye-like structures on the ratio of the height of conical protuberances with the incident wavelength of light (*d*/λ). Adapted with permission from Macmillan Publishers Ltd: Nature [64], copyright 1973.

The height of these conical protuberances (*d*) shares a special relationship with the incident wavelength (λ) of light, which is responsible for developing the anti-reflection property mimicking the moth eye. The principle was first given by Rayleigh. When *d* is extremely small, as compared to λ, the surface is sharp and shows maximum reflectance. As *d* increases, it is accompanied by a decrease in reflectance levels, with a minimum value of zero attained at *d*/λ = 0.4. If we further keep on increasing the height *d* of these conical protuberances, we obtain successive maxima and minima of reflectance levels, none though reaching a value as high as when *d* was extremely small as compared to λ. When *d* reaches a value that is very large as compared to λ, we obtain practically zero reflectance. This behavior has been demonstrated in Figure 13. Thus, to obtain anti-reflecting properties in the visible spectrum, we have to make conical protuberances with a height greater than or equal to 250 nm [64].

**Figure 14:**
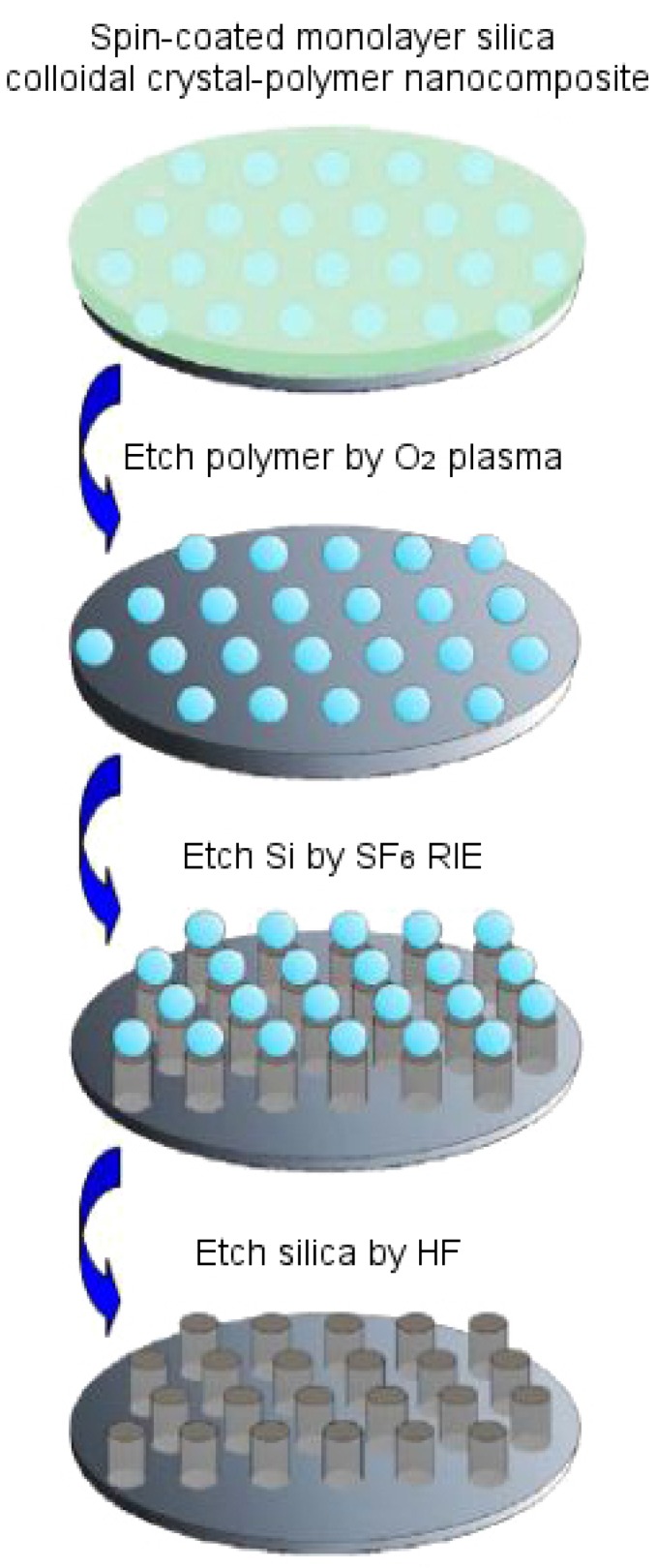
Schematic diagram for fabricating moth eye-like conical protuberances on silicon substrate using the templating technique for anti-reflection properties. Reproduced from [66] with the permission of AIP Publishing. RIE: Reactive ion etching.

To fabricate moth eye-like nanostructures artificially, the soft imprint lithographic technique can be used to generate a negative mold with materials like perfluoropolyether (PFPE), as shown in Figure 12b, which can then be used to generate another positive mold that mimics the morphology of the eye surface. Another method, based on a bioinspired templating technique, allows us to fabricate anti-reflection coatings on single crystalline silicon substrate. In doing this, a silica colloidal crystal-polymer nanocomposite is fabricated using spin coating, in which polymer is etched with oxygen plasma, Si is etched with SF_6_ reactive ion etching (SF_6_ RIE) and silica is etched with hydrofluoric acid to obtain silicon nipple arrays mimicking moth eye [66]. Figure 14 demonstrates this fabrication technique. A slightly altered route is followed by Linn et al. [67] to fabricate similar nanostructures. A third method involves fabrication using the roller nanoimprint technique [16]. Here, a master layer containing nanoscale structures is pressed sequentially on a UV-sensitive resist which has been spin coated on a silicon substrate. This UV-sensitive resist acts as an etching mask when moth-eye like patterns are transferred to the substrate using inductively coupled plasma etching or reactive ion etching to form conical protuberances.

The anti-reflective properties of the bioinspired moth eye-like structures have recently been demonstrated to possess numerous potential applications, for example in eyeglasses, flat-displays, solar panels, etc. They have been shown to increase the efficiency of both perovskite, as well as organic solar cells. In one study, mesoporous TiO_2_ film mimicking moth eye-like nanostructures were prepared using the lithography, nanoimprinting and PDMS stamping technique, which increased the power conversion efficiency by ≈11% as against conventional perovskite solar cells [68]. In another study, organic polymers were used to prepare moth eye-mimicking organic solar cells [69], which are economical and may one day solve our energy crisis by providing solar energy-converted electricity efficiently to our homes and offices. In yet another study, moth eye-based filters were tested for various optical properties, like wavefront quality, transmission and the light scattered, by deploying them in infrared instruments at normal and at cryogenic temperatures for ground- and space-based applications [70].

### 3.6. Butterfly Wings

Nature works in sophisticated ways, and time and again, it is proven by studying it closely. For example, even small insects like butterflies exhibit highly complicated physical properties by possessing unique optical characteristics. The wings of a butterfly demonstrate high or low reflectivity at particular wavelengths of light while simultaneously diffusing reflected light in a wide angular spectrum. These properties are extremely important for their survival via camouflage, for mating and for marking territorial boundaries. Biomimeticians are particularly interested in the iridescence property of butterfly wings, which causes them to change color when viewed from different angles, sometimes even making the wing look metallic [71]. This property is a direct result of the micro- and nano-structural composition of butterfly wings as discussed in subsequent paragraphs.

**Figure 15:**
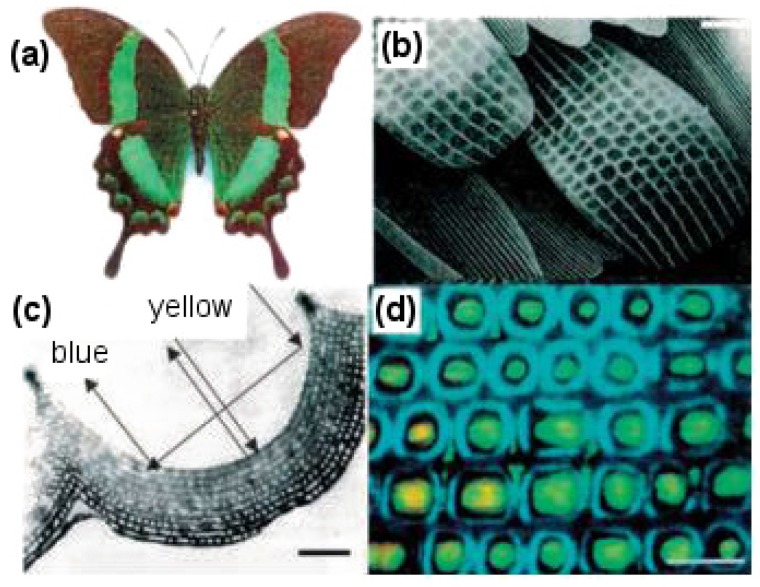
Color-mixing phenomenon displayed by *Papilio palinurus* butterfly wings resulting from the concavities across the surface of the wing scales. (**a**) *P. palinurus* butterfly with a wingspan of 100 mm; (**b**) Scanning electron microscopy (SEM) image showing the overlapping roof tile pattern of scales on the butterfly wing surface; (**c**) Transmission electron microscopy (TEM) image showing a cross section through one concavity on a *P. palinurus* butterfly scale and color-mixing mechanism; (**d**) Optical micrograph showing repeated structures of lamellae and reticulum forming arrays to display colors [72]. Scale bars: (**b**) 5 μm, (**c**) 1 μm, and (**d**) 10 μm.

Butterflies are of particular interest for studying nanostructures because they have a rich diversity of scales coating their wings that bring about beautiful colors and patterns (see Figure 15). The arrangement of scales follows an overlapping roof tile pattern with a scale density of 200–600 per mm^2^ depending on the species. Apart from the simple roof tile pattern, scales further possess voids, complicated groove shapes and stratification, which allow them to display complex optical effects, like light scattering, diffraction and interference [72]. It is interesting to note that color is usually linked with chromophore pigments, which are responsible for chemically displaying the color, but in the case of butterfly wings, various optical phenomena of light taking place on physically-patterned scales are responsible for color generation [2] (see Figure 15c).

By looking more closely at these scales, it can be observed that on a single wing, they are present in the thousands and have dimensions of about 200 μm in length and 50 μm in width. The wings consist of two sets of scales with an alternate row arrangement, a set of long scales acting as covers, which overlap and hide a set of shorter ground-level scales. A raised grid of longitudinal quasi-parallel ridges (lamellae) constituting the upper surface of the scale run along its complete length with a spacing of about 2.5 μm. Fine tubes forming a net-like latticework (reticulum) cover the spacing between two adjacent lamellae. These lamellae and reticulum, forming arrays of repeated structures, work together to provide the observed characteristic colors of butterfly wings.

To fabricate butterfly wing-like structures in the lab, several techniques have been reported in the literature. Conformal evaporated film by rotation (CEFR) is one such specialized physical vapor deposition method [73]. It involves thermal evaporation of chalcogenide glasses constituted of materials like GeSbSe, to form collimated vapor flux in a low pressure chamber, which provides a covering over a biotemplate. The biotemplate is rotated in a complex manner such that vapor flux material forms a uniform coating on its surface. Glass composition of GeSbSe is selected due to its excellent optical and mechanical properties. Another method used for fabricating artificial butterfly wings is a three-step dipping process [74]. In doing this, wings undergo an initial pre-treatment process, after which they are dipped in a closed vessel-containing precursor solution of zinc oxide. They are then taken out, dried and further treated at high temperature to remove the wing template and obtain a ceramic surface morphology mimicking the butterfly wings.

Other methods used to fabricate artificial butterfly wings include deposition of silicate minerals in the inner spaces of wing scales by chemical vapor deposition to form a thick coating of about 100–150 nm on its surface, to obtain silicified butterfly wings [75]. The sol–gel technique to fabricate highly ordered structures of lead lanthanum zirconate titanate (PLZT) mimicking butterfly wings uses dip coating [76]. Titanium dioxide-, tin dioxide (SnO_2_)- and silicon dioxide (SiO_2_)-based wing morphology uses the sonochemical method followed by a calcination process [77]. Butterfly wings react readily to external stimuli, like temperature and humidity. Needless to say, biomimetic structures inspired by butterfly wings have recently found application in a number of sensors, like temperature and infrared sensors, vapor and solvent sensors and pH sensors [78].

### 3.7. Frog Toe Pads

The adhesive systems used by various animals have intrigued researchers to investigate the dynamic mechanisms of adhesion. Studies show that the reason adhesive forces occur is due to a bond formed between the animal and the surface, which opposes the gravitational pull of the Earth [79]. In Section 3.2, we already saw the dual characteristic of superhydrophobicity and the high adhesivity of gecko feet towards wet surfaces. However, other structural adaptations need to be studied as adhesion in animals can occur in multiple ways. In fact, there are four different adhesion mechanisms that we can observe in animals, namely gluing, suction, wet adhesion and dry adhesion [80]. Echinoderms (sea urchins and starfish) use gluing for attachment and locomotion, whereas suction is used by disk-winged bats by reducing internal pressure. In dry adhesion, the formation of intermolecular/van der Waals forces between the animal and the surface leads to adhesion. As a result, geckos have the ability to climb smooth vertical surfaces and run on ceilings. Wet adhesion observed in amphibians, ants and grasshoppers results from the combined forces of surface tension and viscosity produced by the fluid layer between the pad and the substrate.

**Figure 16:**
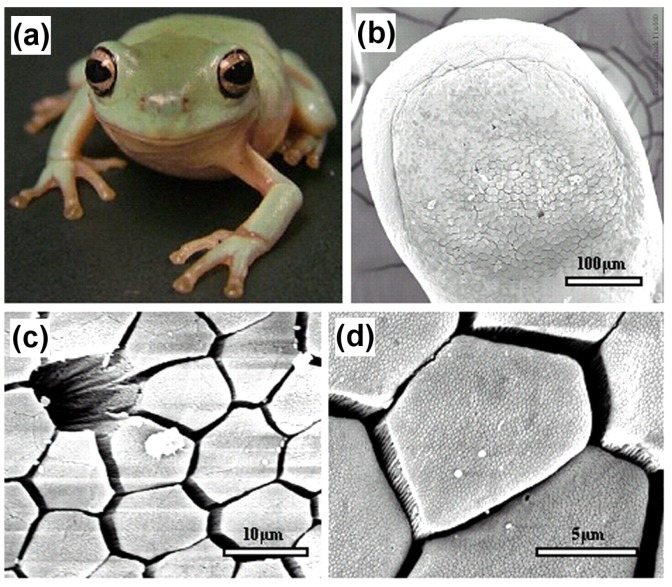
Adhesive surface of the frog toe pad. (**a**) Immature White’s tree frog (*Litoria caerulea*) with snout to vent length of approximately 40 mm; (**b**) Low resolution scanning electron microscopy (SEM) image of the whole pad of a juvenile frog; (**c**) Medium resolution SEM image showing the mucous pore and (mainly) hexagonal epithelial cells separated by channels at their distal ends; (**d**) High resolution SEM image showing the presence of nanostructures on the surface of the epithelial cells [81]. Reproduced with permission from The Journal of Experimental Biology.

In amphibians, adhesive toe pads can be found in seven families of frogs, including torrent or tree frogs and arboreal salamanders. The toe pads are comprised of columnar epithelial cells with regular hexagonal microstructures of about 10 μm in diameter. These are separated from each other at their tips by 1 μm-wide channels [81]. Figure 16 depicts the hexagonal microstructures and separation channels. The channels consist of mucous-secreating glands, forming a thin layer between the pad and the substrate. Earlier researchers presumed that mucous acts as glue with which the animal is able to stick itself to the substrate [82]. After years of intensive research, it was evident that a visible meniscus lies at the area of contact between the pad and the substrate [83], and the shear forces of toe pad responsible for adhesion were also found to be velocity dependent [84]. Further, the sticking ability of frogs was observed to decrease in the case of wet toes [85], but at the same time, fresh water frogs demonstrated capabilities to climb on wet rocks with water flowing over the substrate [86].

**Figure 17:**
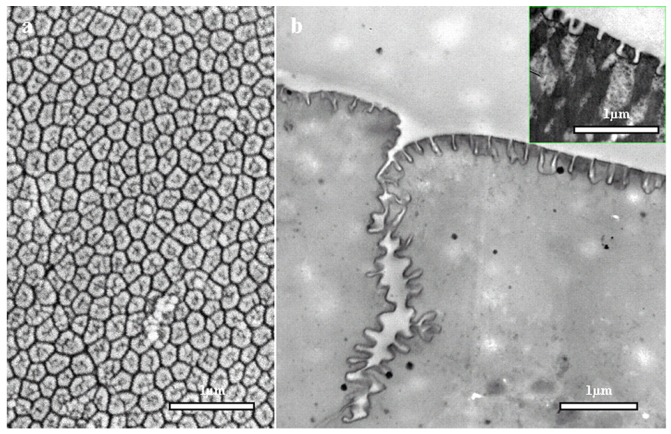
Nanostructural features of the frog toe pad. (**a**) High resolution scanning electron microscopy (SEM) image of a large number of hexagonal nanostructures forming an array on the external surface of the epithelial cell; (**b**) High resolution transmission electron microscopy (TEM) image of one of the channels that separates adjacent epithelial cells along with the side view of nanostructures, which themselves are separated by even narrower channels. The inset shows similar nanostructures on the toe pad of the hylid tree frog *Scinax rubber* [81]. Reproduced with permission from The Journal of Experimental Biology.

With the recent development of microscopic techniques like SEM, transmission electron microscopy (TEM) and AFM, it has been found that the top of the toe pad epithelial cells is not flat, but comprised of a dense array of nanopillars, which are 300–400 nm both in diameter and height. These nanopillars come in direct contact with the substrate producing considerable shear force and are responsible for the adhesive characteristic of the frog toe pad via wet adhesion. Figure 17 illustrates the nanopillars resting on top of hexagonal microstructures along with a higher magnification view of the separation channels.

**Figure 18:**
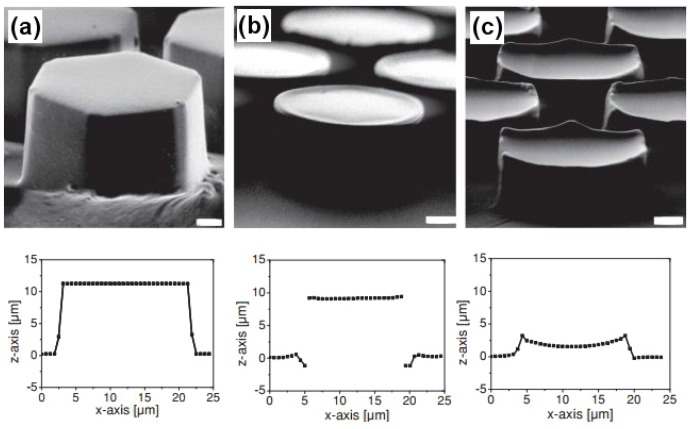
Biomimetic hexagonal surface textures. Scanning electron microscopy (SEM) images of polydimethylsiloxane (PDMS) arrays of hexagonal micropillars (15–19 μm diameter) terminated with (**a**) flat tips, (**b**) T-shaped tips (10 μm height, 3 and 5 μm channel width) and (**c**) concave tips (3 μm height, 5 μm channel width). Scale bars correspond to 2 μm. Below, the corresponding profiles of pillars as obtained with confocal microscope are shown [87].

Not much has been done to mimic the frog toe pad structure, partly due to its complicated design, which needs to be studied further in order to be fabricated in the lab. However, in one study similar structures were fabricated having different microstructured surface designs using photolithography [87]. Polydimethylsiloxane being a soft elastomer and with a Young’s modulus of 2 MPa lying in close proximity with the frog toe pad (∼5 MPa) was the preferred choice as the fabricating material [81]. Figure 18 demonstrates the hexagonal micropillared arrays with flat, T-shaped and concave surface designs along with their dimensional profiles obtained using confocal microscopy. In more recent studies, the frog toe pad structure has been used to inspire the design of safety razors [88] and surgical graspers [89]. Polyvinyl siloxane (PVS) was the preferred material choice for designing the hexagonal toe pad-like surface texture of safety razors. The razors thus developed gave almost double sliding friction as against the presently available disposable safety razors when tested on lubricated human skin. In the case of the surgical grasper, hexagonal micropillared patterns fabricated using PDMS were compared to the jaw of currently sold surgical graspers. The biomimetic structure exhibited stronger and more stable friction on the surface of soft tissue while incurring minimal damage or deformation of tissue.

## 4. Future Perspectives and Applications of Biomimetics

It is fascinating to think about the future with many bioinspired materials going from the lab scale to bulk production for practical applications. Superhydrophobic materials fabricated by studying the lotus leaf will find potential applications in automobile glasses, building windows, textiles, paints and emulsions. Materials inspired by the dual characteristics of gecko feet can be used as mechanical hands for separating a water droplet from a less adhesive superhydrophobic surface [90]. Being adhesive to wet surfaces, these have also influenced the design of biodegradable bandages, which tend to solve the problem of bandage fixation on wet and moving tissues [91,92]. Another important application is the use of dry adhesive treads inspired from gecko feet in wall climbing robots [93].

Self-healing materials ensure more sturdy and durable construction materials for infrastructure, as well as space applications. They also provide improved defense materials as in the case of body armor. Successfully mimicking the process of photosynthesis will have many potential applications, like in sensor design, hydrogen fuel generation by water splitting, molecular level optoelectronics and photonics. Hydrogen is regarded as the fuel of tomorrow, and with artificial photosynthesis, hydrogen can be generated by splitting water and producing oxygen as a by-product. Developing this technology as quickly as possible to work efficiently will prove to be a real gift for mankind, eliminating our dependence on exhaustive fuels and preventing environmental pollution. Moth eye increases our understanding about anti-reflecting surfaces. Solar cells currently reflect back 30% of absorbed light. An anti-reflecting coating on their surface will tremendously increase their efficiency. Moreover anti-reflection coatings over automobile glasses, windows and everyday use glassware will provide their own unique advantages.

Bioinspired artificial butterfly wings can be used as photonic crystals, i.e., optical materials with distinctive features. These will find potential applications in color-changing inks, paints, glasses, high or low reflectivity surfaces and materials possessing light confinement properties. Due to such novel characteristics, these crystals can be used as building material for nano- and microscale devices, like UV light-emitting diodes. Frog toe pads are widely researched for their application in tire treads. Their hexagonal microstructures have been mimicked on tires [94]. Tires have also been designed with different patterns, for example channels mimicking frog toe pad nanostructures have been placed in between tire treads. These channels provide a solid grip on wet roads, by allowing water/snow to flow through these channels, resulting in an intimate surface to tire contact. Another application can be found in the design of non-slippery footwear.

These are some of the numerous applications that will owe their origin to the field of biomimetics. A few years down the line, artificially fabricated bioinspired nanostructures will be implemented in more and more products to obtain novel applications. These materials are shaping our future in a very exciting way.

## 5. Conclusions

In this review, we focused on how nanoscience and nanotechnology proved to be a boon for carrying out advancements in the field of biomimetics. It is evident how important it was to look closely at nanostructures present in nature to successfully mimic them in the lab. This was only made possible by the development of various microscopic and spectroscopic tools for nanotechnology. We then discussed the nanostructures of lotus leaf, gecko feet, moth eye, butterfly wings and frog toe pads and studied the natural processes of biological wound healing and photosynthesis. Along with this, for each type of nanostructure and natural process, the techniques used for their artificial fabrication were also discussed. Then, some of the novel applications of these bioinspired materials have been mentioned and how these are changing the shape of our future. Finally, it is important to emphasize that nature will always keep on surprising us, if we keep on looking closely and understand its working. We should be continuously on the lookout to study more natural processes and structures to develop more unique materials that can be life-changing. For example, we have not yet been able to synthesize silk artificially in the lab without involving spiders or silkworms. Then, as is always said, nature does it best.
